# Recurrent outbreaks of mumps in Lothian and the impact of waning immunity

**DOI:** 10.1017/S0950268820001296

**Published:** 2020-06-18

**Authors:** C. J. Waugh, L. J. Willocks, K. Templeton, J. Stevenson

**Affiliations:** 1 NHS National Services Scotland, Edinburgh, UK; 2 NHS Lothian, Edinburgh, UK

**Keywords:** Community outbreaks, infectious disease epidemiology, mumps, MMR vaccination, immunization (vaccination)

## Abstract

Another large outbreak of mumps occurred in Lothian from October 2017, which coincided with the commencement of the higher education term. During this period 324 cases were notified, most of whom were aged 18–22 years old. Although previous outbreaks had a focus in student populations, 43% of current cases reported that they were not a student. There has been increases in private student housing where students from all universities live, which may have contributed to the wide spread of the outbreak and complicated outbreak control. Information on vaccination status was available for 244 cases (75%), of whom the majority (75.8%) reported having two MMR doses. To investigate potential waning vaccine immunity the mean length of time since last mumps containing vaccine was calculated as 14.3 years. The outbreak was declared over in May 2018 after case numbers returned to background levels. This outbreak highlighted that mumps outbreaks occur cyclically coinciding with new cohorts of susceptible students entering the Lothian population. The lessons from this outbreak are to encourage students to have two MMR doses and also be prepared for mumps outbreaks in the near future. In future outbreaks the utility of a third MMR for outbreak control could be examined.

## Introduction

Outbreaks of mumps are increasingly being reported in young people, especially among student populations [1–7]. Many of these outbreaks are ascribed to low MMR coverage [7].

**Fig. 1.**
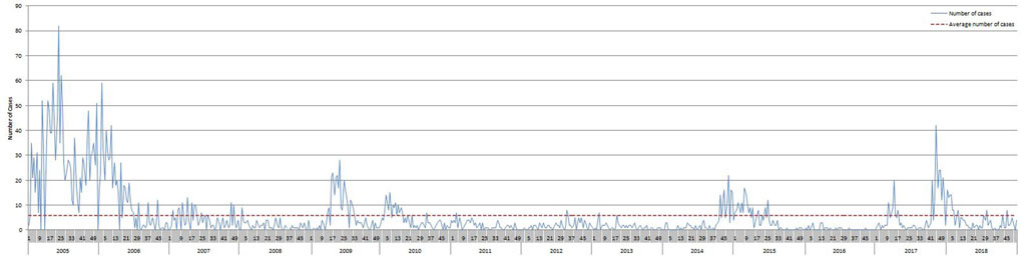
Epidemic curve of mumps cases in Lothian 2007–2018.

In Lothian, Scotland, we have noticed a cyclical pattern of mumps outbreaks in Lothian every three to four years (Fig. 1). Mumps outbreaks associated with higher education settings were seen in 2007, 2009 and 2014. The outbreaks prior to 2014 were predominantly in undervaccinated populations [8, 9]. By contrast, we reported a large outbreak of mumps among students during academic year 2014/2015 and found this to be a highly vaccinated population [9]. Of the 278 cases (of total 341) where vaccination status was available, 84% had received at least one dose of mumps containing vaccine and 62% had received two.

We now report a further mumps outbreak in Lothian during academic year 2017/2018 which we investigated to determine if this was also in a highly vaccinated population and specifically to assess time since MMR vaccination.

### Setting

Lothian in south east Scotland comprises Edinburgh and the surrounding area (population 850 000). There are four universities and the area has a large multi-cultural student population of over 60 000 [9].

Since 1996 the UK MMR vaccination programme has comprised two doses, the first at 12–15 months of age and the second dose is given preschool. Uptake in Scotland has consistently been high with 91.2% percentage of children receiving two doses of vaccine by the age of 5 [10]. Even at the nadir of uptake after the Wakefield scandal, uptake fell no lower than 82.4% (2007) [11].

## Methods

The definition of a case used throughout this outbreak included all notifications of mumps; both laboratory confirmed and clinical diagnoses.

Laboratory confirmed cases were notified electronically direct from the laboratory management system. All cases submitted to the laboratory were performed by real-time PCR. Clinical notifications were received from general practitioners by phone, by email or through the electronic notification system.

A surveillance questionnaire was sent to each case to try to ascertain whether they were a student, what their vaccination status was and what medical attention they had required including information on their symptoms and any complications they had experienced. Futher information on vaccination status was obtained from surveillance forms, calling GP practices and from Scottish Immunisation Recall System (SIRS). Information on dates of vaccination was taken from SIRS.

A dataset was compiled for each case combining information from all the above data sources.

## Results

### Time

**Fig. 2.**
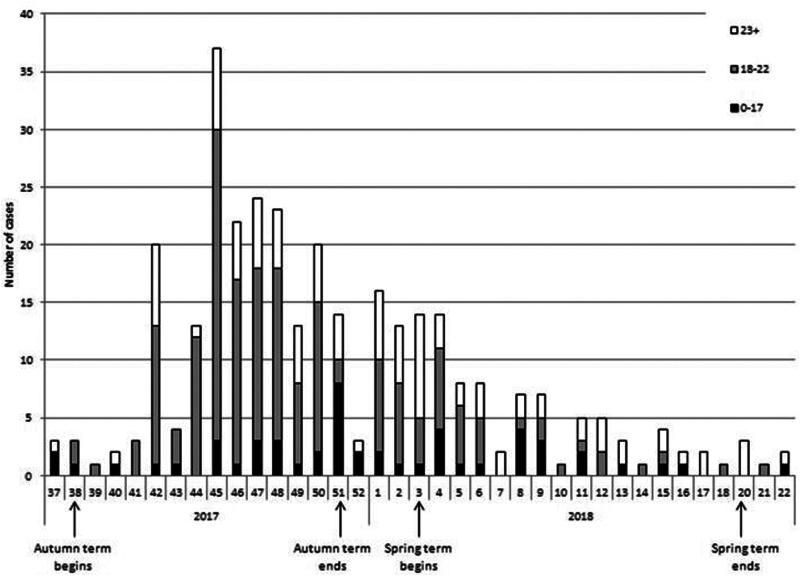
Epidemic curve of mumps cases by age group (*n* = 324), by week.

The outbreak began in week 42 (October 2017) shortly after the start of the academic year on 18 September 2017. There was an increase in notifications 4 weeks after the start of the Autumn term as can be seen in Figure 2. The outbreak was declared over in May 2018 by which time 324 cases were recorded.

### Person

The majority (53.4%) of cases were between the ages of 18 and 22 years old (Table 1). The 13–17 and 23–27 age groups accounted for 11.4% and 11.7% of cases respectively. Those over 38 years old made up 6.2% of all cases. The figure below shows the changes in age groups over the duration of the outbreak. There was a wide range in age of cases from those under one year old up to a case aged 73 years old. There were slightly more male cases reported during this outbreak with male cases making up 54.3% of all the cases.

**Table 1.** Number of student cases by higher education institute

Student status	Number of cases	Percentage of cases (%)
Institute A	19	5.9
Institute B	32	9.9
Institute C	11	3.4
Institute D	5	1.5
Institute E	1	0.3
Unknown Institute	10	3.1
Institute Out with Lothian	8	2.5
Non-Student	105	32.4
Working in Higher Education	5	1.5
School Pupil	30	9.3
Unknown Student Status	98	30.3

### Place

**Fig. 3.**
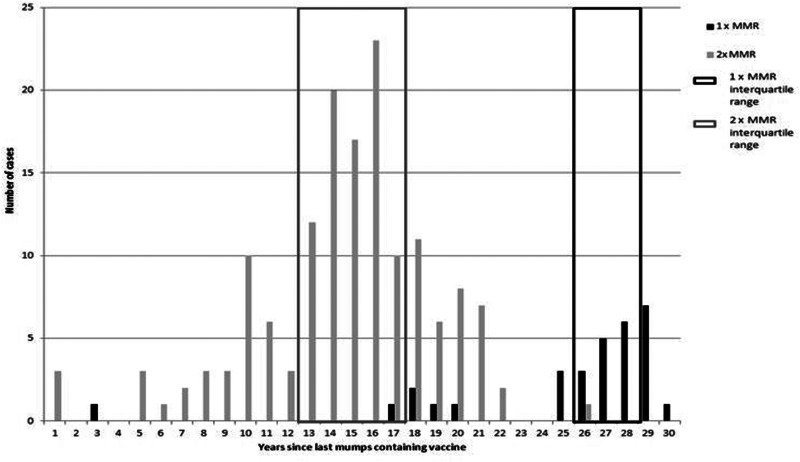
Years since last mumps containing vaccine and number of doses (*n* = 181).

When the proportion of cases recorded as attending a higher education institute was examined it was found that a large proportion of cases (43.2%) were not associated with any of these institutes. There were number of cases that did not have a student status ascertained (30.3%). A number of cases (68) identified themselves as students at a higher education institute. All universities in Lothian had at least one case of mumps; however Institute B experienced the highest number of cases with 32 cases being notified between September 2017 and May 2018 (Fig. 3). Eight cases reported attending universities out with the Lothian area. A small number of cases reported they were members of staff at a higher education institute.

Of the 98 cases who were classified as of unknown student status 53 were aged 18–22 which could represent underreporting of students status.

There were 30 cases in school pupils with there being small clusters of cases in five different schools. There was also a cluster of cases associated with a sports team.

### Morbidity

Completed surveillance forms were returned for 148 (45.7%) cases.

Of those cases completing the surveillance form 129 out of 148 (87.2%) reported that they had been to the GP and five cases (3.4%) reported having attended hospital, with one case having reported being admitted with a length of stay of five days. There were seven reported cases of orchitis (4.7%). One case described some hearing loss.

### Vaccination status

Information on the vaccination status was obtained for 244 cases (75.3%). Of the 244 where status was known 185 cases had had two doses of MMR (76%) and 35 cases (14.3%) had only one MMR. There were no MMRs recorded for 24 cases (9.8%).

Cases that were not born in Lothian were unlikely to be on SIRS; for 58 cases (17.9%) there was no available record on the system. Some cases reported having vaccinations in other countries, some were either too young to have begun the vaccination programme or were too old to be eligible for MMR when this programme was launched Table 2.

**Table 2.** Mumps cases by MMR status and age group (*n* = 324)

MMR status	0–12	13–17	18–22	23–27	28–32	33–37	38 +	Unknown	Grand total
1 × MMR	2	2	5	9	14	3			35
2 × MMR	11	33	113	21	6		1		185
No MMR	1	1	16	1	1	3	1		24
Not on SIRS		1	37	6	7	4	3		58
Other vaccine regimens			1		1	1			3
Out with age range	1						15		16
Unknown			2					1	3
Grand Total	15	37	174	37	29	11	20	1	324

For those in the age group most affected, 18–22 years old, of the 134 where vaccination status was known 112 (84%) had received two doses of MMR. It is interesting to note that 33 out of the 37 13–17 year olds affected had two documented MMRs.

### Time since MMR

In 181 records data were available on the date of MMR vaccination. The mean length of time since last mumps containing vaccine was 14.3 years (range 4–30 years). The length of time since last mumps containing vaccine was examined by age group (Table 3). In the predominant age group (18–22) most cases had not had a mumps containing vaccine for 15.3 years (18.7 years for those with only one dose of MMR). The interquartile range for those with only one dose of MMR is higher than that of those with two doses (Figs 3). This highlights that for most cases those with only one dose of MMR also had a longer time without a dose of mumps containing vaccine.

**Table 3.** Time since last dose of mumps containing vaccine in years by age group.

MMR Status	0–12	13–17	18–22	23–27	28–32	33–37	38+
1 × MMR	3.0	9.0	18.7	26.3	27.8	29.5	N/A*
2 × MMR	8.0	12.0	15.3	20.4	15.0	N/A*	17.0

*Refer to Table 1.

### Outbreak measures

Control measures proved difficult to implement during this outbreak for a number of reasons. During this outbreak an alert was sent to GPs to increase awareness of the condition alongside targeted health messages were sent jointly with universities and were distributed to the university halls of residence. This was difficult to replicate with the private facilities as there was neither a key point of contact nor a complete list of all the facilities in our area. The increase in the number of private student accommodations in Edinburgh meant that there was a potential for the outbreak to spread between the higher education institutes than has been seen during previous outbreaks. Due to the large geographical area these cases covered it was felt that any outbreak control measure using vaccination would not be feasible. The outbreak spanned the Christmas break in universities which may have lead to a lower return rate of surveillance forms and also posed a risk of cases being missed as part of this outbreak if identified in other areas during the break.

## Discussion

This paper summarises the epidemiology of a large outbreak of 324 cases of mumps in Lothian between September 2017 and May 2018. Surveillance of cases identified that a large proportion of mumps cases were aged 18–22 years, were fully vaccinated with two doses of MMR and had a mild pattern of illness.

This outbreak highlights that mumps infections can occur even in individuals who have had two doses of MMR, particularly when they are aged 18–22 years. The cyclical pattern of outbreaks in Lothian seems to mirror what has been reported by Public health England that there are periodic outbreaks of mumps infections every three to four years [12]. This could be due to the build up of a new cohort of susceptible people of that age group in the area. [13]. Of those with immunisation data 75.8% had two doses of MMR recorded. According to ISD statistic Lothian has a completion rate of two doses of MMR of 91.2% by the age of five, both of these vaccination levels fall below the WHO recommended level of 95% coverage which would help reduce the risk of outbreaks overall [10]. Further research is required to establish the reason behind these outbreaks and the impact of waning immunity [14].

The mean length of time since cases with available information had a mumps containing vaccine was 14.3 years; however this did range from 4 years up to 29 years. This is interesting as a recent model examining waning immunity found that it would be expected for 25% of people to be susceptible to infection after 7.9 years, 50% of people to be susceptible after 19 years and 75% of people to be susceptible after 38 years since their last mumps containing vaccine [1]. During an outbreak at a University in 2015 it was also noted that if a student had received a second dose of MMR longer than 13 years ago then the attack rate was significantly higher, which matches the findings during the most recent Lothian outbreak [15]. Our findings are further supported as during an outbreak in France the mean time from the last dose to symptom onset was reported as 13 years [2]. It has also been suggested that waning immunity could lead to periodic outbreaks which supports the cyclical nature of the epicurve of mumps cases over time [16].

This study is strengthened by the availability of up to date immunisation data that allows us to examine the time since last mumps containing vaccine. It would be useful for future epidemiological studies and or outbreak management if Universities were to hold records of their students' vaccination status to allow for a more complete vaccination status of cases to be examined.

This 2017–2018 mumps outbreak was wide spread in the community and not just in higher education settings so did not allow the investigation of the effectiveness of a third MMR as a control measure during an outbreak. The impact of a third MMR as a control measure during outbreaks in populations has been examined in specific outbreaks and has been demonstrated to reduce the attack rate in these groups [15, 17]. However it has also been reported that a third dose of MMR has little prophylactic effect post exposure [3]. If there was to be another outbreak of mumps, which appears likely given the local epidemiology, consideration should be given to setting up a study to evaluate the effectiveness of a third MMR for the control of a mumps outbreak.

In 2017–2018 cases were reported at different universities and cases were also seen in the general population with no obvious links to higher education. This differs from the mumps outbreak in 2014 where there were a high number of the cases associated with a specific university which then seeded to other universities and then the general public [9]. However, both the 2014 and 2017 outbreaks did coincide with the commencement of the autumn academic year when large numbers of new students arrive in the city. Many students in Edinburgh now live in private student housing where students from all universities live in close proximity and mix.

This outbreak had a more explosive start than the previous outbreak in 2014 but there were a lower overall number of cases [9]. The periodic occurrence of mumps outbreaks raises questions around whether the current two dose MMR vaccination programme completed in childhood leads to waning immunity to the virus by late teenage years and whether a booster may be required, especially for students entering higher education [1, 2].

During the outbreak in 2014 genotyping of the mumps samples was undertaken to allow examination of the circulating strain [9]. Unfortunately this was not performed for this study which limits our ability to distinguish between waning immunity and a mismatch between the strain contained in the MMR vaccine and the circulating strain [18].

Examining the deprivation status of those who developed mumps may have provided insight into areas where messages promoting vaccine uptake could be targeted. A recent study has shown that those in the most deprived socioeconomic groups there is a delay in uptake and also a delay in the timeliness of vaccination which could impact on the effects of waning immunity [19]. This may, however, have proven difficult with a large number of the cases reporting as student. In future outbreak an examination of this in resident cases may prove to be insightful.

There have been a number of reports of universities being the focal point for outbreaks of mumps around the world [2, 4, 5]. A study evaluating outbreaks between 2010 and 2015 in the United States found that 78% of all of the outbreaks were at universities [6]. The 18–22 year old age group remains susceptible to measles and mumps infection [9, 20]. It is important that messages around the value of two doses of MMR continue to be reinforced especially for new students arriving from around the world. A status of two doses of MMR should be confirmed and immunizations completed if required before students arrive at university. Two doses of MMR would not only boost immunity to mumps but would also ensure protection against measles.
